# Prevalence of insufficient daily physical activity and its association with health indicators among Chinese primary and secondary school students: a cross-sectional study

**DOI:** 10.3389/fpubh.2026.1732510

**Published:** 2026-02-04

**Authors:** Rui Qin, Jingtao Wu, Wanli Zang, Dong Zhang, Xiaowei Feng

**Affiliations:** 1 School of Sports Medicine and Health, Chengdu Institute of Physical Education, Chengdu, China; 2 School of Physical Education, Leshan Normal University, Leshan, China; 3 Postgraduate School, Harbin Sport University, Harbin, China; 4 College of Physical Education, Quanzhou Normal University, Quanzhou, Fujian, China; 5 School of Football, Hainan Normal University, Haikou, China

**Keywords:** academic stress, health cognition, physical activity, public health policy, risk factors, youth

## Abstract

**Objective:**

Based on an epidemiological investigation of 2-h of daily physical activity among primary and secondary school students across China's seven major administrative regions, this study analyzes the association between social support and cognitive factors, thereby providing recommendations for policy formulation.

**Methods:**

A cross-sectional study was conducted using random cluster sampling across seven Chinese administrative regions from May to July 2025. Data were collected using standardized scales measuring physical activity levels, achievement of the daily 2-h physical activity target, academic stress, and health cognition. Statistical analyses, including descriptive statistics, chi-square tests, and multivariable logistic regression, were performed using *SPSS* 26.0.

**Results:**

(1) The prevalence of insufficient 2-h daily physical activity showed significant differences across various demographic variables (all *p* < 0.001). Higher prevalence rates were observed among students in rural areas (30.50%), private schools (29.41%), boarding students (30.03%), and those with lower frequency of reunions with parents. (2) Key risk factors identified included lack of health cognition, low perceived value of physical activity, lack of sports facilities, insufficient allocated physical education time, and lack of access to smart devices. (3) Significant urban-rural/regional disparities were found regarding access to smart devices (*t* = 3.142, *p* = 0.002) and academic stress levels (*t* = 2.499, *p* = 0.012).

**Conclusion:**

Health cognition, resource availability, and time allocation are significant factors associated with the insufficiency of daily 2-h physical activity among Chinese primary and secondary school students. The education department has increased the guarantee of student system construction, and has implemented differentiated management for higher grades.

## 1 Introduction

Since the turn of the new century, rates of overweight/obesity and poor eyesight have been on the rise among primary and secondary school students in China while indicators of physical fitness such as explosive strength have shown a downward trend. It is estimated that there are about 12% of children in China who are overweight, and among the obese primary school students, 45%−50% will remain obese when they become adults, and among the obese secondary school students, 60%−70% will remain obese when they become adults (1). Physical inactivity is one of the main causes of overweight and obesity in adolescents and a high-risk factor for the prevalence of obesity and chronic cardiovascular diseases (2). According to the definition of World Health Organization (WHO), physical inactivity is a global non-communicable disease prevalent in both developed and developing countries (3). Most of the scholars believe that there is a significant correlation between the level of physical activity and various health risk factors (4, 5).

According to the outline of Construction Plan of China Education Powerhouse (2024–2035) issued by Ministry of Education of China, it is clearly stipulated that “comprehensive physical activity time for primary and secondary school students should not be less than 2 h per day” (6). Scholars also pointed out that physical inactivity is common among Chinese students and there is a relatively large gap to the above “2-h” target (7). The gap between the current situation and target has become a major public health issue that need to be paid attention to.

Although Global Status Report on Physical Activity (2022) pointed out that physical inactivity is a problem for all ages worldwide, especially for children, adolescents and the older adults (8), systematic research on the epidemiological characteristics of “insufficient 2-h physical activity per day” and its spectrum of health risks are still relatively lacking (9).

In view of the above situations, this study intends to carry out a nationwide cross-sectional survey and tries to systematically describe the epidemiological distribution characteristics of “insufficient 2-h physical activity per day” among primary and secondary school students in China and quantitatively explore its correlation with overweight/obesity, poor eyesight and explosive strength, so as to provide a scientific basis for the formulation of policies and intervention measures.

### 1.1 The epidemiology of physical inactivity in children and adolescents: a public health perspective

According to the survey results of World Health Organization (WHO), more than 80% of school-attending adolescents around the world do not meet the recommendation of at least 1 h of physical activity per day (10). There are socioeconomic disparities in children and adolescents' participation in overall physical activities; disparities in children and adolescents' participation in organized sports are greater than those in total participation and are larger between children and adolescents (11). This phenomenon exists in all income countries and regions: the prevalence of physical inactivity is 84.9% in low-income countries, approaching 80% in high-income countries, highest globally in the Asia-Pacific region (girls 95.6%, boys 89.0%), and lowest in boys in high-income Western countries (72.1%) (12).

This phenomenon exists in all income countries and regions and all cultural contexts: it is a widespread phenomenon reflecting the persistent decline in activity levels among adolescents amidst modern society and society, new type of education, and lifestyle (13). In the four decades since China's reform and opening-up, it has encountered a public health problem during the process of socio-demographic transition and lifestyle changes. The rate of adolescent obesity quadrupled, but the proportion of adolescents meeting the recommendations of physical activity guidelines remains very low, which is a serious situation. Improving the overall health level of more than 170 million children and adolescents in our country is extremely urgent (1).

Longitudinal cohort studies and multiple systematic reviews have linked physical inactivity in children and adolescents to the high risk of development of obesity and several chronic cardiovascular diseases and have shown significant associations with metabolic syndrome, bone health, and mental health (14). With respect to physical inactivity, school-based interventions can significantly influence the physical activity and health status of children and adolescents (15). In this comprehensive review, Heath et al. (16) analyzed and systematically summarized the dose-response effects of numerous different types of physical activity interventions (not limited to children and adolescents). In scholars' opinions, although a large volume of research focused on adolescents exists, much more can be done to close the gaps in knowledge. It is essential to strengthen the monitoring of adolescent physical activity, implementation of interventions, and policy development (17).

### 1.2 Health risks of physical inactivity: physiological, psychological, and academic evidence

Physical inactivity is prevalent among children and adolescents worldwide and is now the biggest health threat to children and adolescents worldwide (18). Physical activity can prevent diseases and delay the premature onset of unhealthy conditions such as obesity, coronary heart disease and other diseases (19). Apart from the lack of physical activity, another highly controversial and different form of lifestyle — “sedentary behavior” is also regarded as a cause of poor health (8). Sedentary behaviors may induce cardiovascular changes through promoting blood pressure, cholesterol and heart disease (20). Sedentary behavior is likely to lead to the development of diabetes and low bone density in adulthood (21).

In the revised WHO 2020 guidelines, sitting time in children aged 5 years and older have strong recommendations that more sitting time, especially recreational screen time, is associated with adverse health consequences (22). Sedentary behaviors will make an intake more than expenditure, leading to body fat accumulation and obesity. Obese children will be more likely to develop cognitive problems, depression, anxiety and other anxiety symptoms (4).

Over the past two decades, many scholars have explored the effects of physical activity on children and adolescents' cognitive function and academic performance. Vorkapic believed that physical activity intervention teaching methods with the participation of team game variations and coordinative activities can improve children's cognitive functions (23). When adolescents are physically active, it can reduce the impact of stressful events on stress symptoms and stimulate the initiative for physical activity, and therefore play the role of a stress buffer in promoting the role of stress buffers (24). It is evident that systematic reviews and meta-analysis also support the role of physical activity in brain development in children, improving executive functions (such as working memory and cognitive flexibility), academic performance, and overall improving children's health-related quality of life (25).

Physical activity has been found to be one of the most important non-pharmacological interventions to mitigate obesity and improve mental health. There is substantial evidence that reduced physical activity leads to a reduced metabolic rate and/or increased food intake, resulting in a positive energy balance, and consequently leading to obesity (26). More importantly, physical activity has positive effects on mental health. Recent studies have reported that exercise can alleviate behavioral and emotional problems in adolescents (27). Haverkamp et al. (28) concluded that both acute and chronic physical activity interventions are effective means to improve various cognitive functions (including verbal skills) and promote academic achievement in adolescent populations. Therefore, physical activity has significant positive effects on the physical health, mental wellbeing, and academic performance of children and adolescents with overweight and obesity.

### 1.3 Research gaps

The WHO and many national health agencies have issued guidelines recommending specific levels and patterns of physical activity for different populations. However, a relatively high proportion of the global population fails to meet these recommendations (29). This is particularly true in China, where the actual implementation status of the “2-h” standard lacks comprehensive, systematic nationwide data. Existing research predominantly focuses on monitoring and analyzing the achievement of the “≥1 h per day” target or general physical inactivity (30), lacking detailed epidemiological description of the attainment rate for the “2 h per day” standard (7, 9).

Most existing studies are confined to single provinces, municipalities, or localized areas, suffering from limited sample representativeness. For instance, some studies on physical inactivity in children only cover China's Inner Mongolia Autonomous Region, making it difficult to reflect variation patterns across different regions, urban/rural settings, genders, school stages, and socioeconomic backgrounds nationwide (31). Furthermore, there is a lack of systematic comparison of disparities between eastern, central, and western regions, and between urban and rural areas, as well as trends in physical inactivity across different school stages (primary, junior high, senior high), which hampers the precise formulation of intervention strategies.

Additionally, quantitative evidence linking physical inactivity relative to this standard with multidimensional health outcomes (e.g., physiological health, mental health, academic performance) remains scarce (19), and there is a lack of cross-sectional studies based on nationally representative Chinese samples.

Therefore, this study will target primary and secondary school students from multiple regions across China. It aims to, for the first time, delineate the epidemiological characteristics of “insufficient daily 2-h physical activity” and systematically assess its correlations with multidimensional health indicators. By incorporating demographic stratification analysis and exploring environmental factors, the study seeks to provide targeted empirical evidence to inform physical activity promotion policies and public health interventions for children and adolescents in China.

## 2 Research procedures and methods

This study employed a cross-sectional design utilizing baseline survey data. A questionnaire survey was conducted from May to July 2025 across seven major administrative regions in mainland China, targeting students from primary school grade one to senior vocational high school levels. This study adopted a stratified multi-stage sampling approach. China was divided into seven major administrative regions (South China, North China, Northwest China, Southwest China, Northeast China, Central China, and East China). Within each region, five prefecture-level cities were randomly selected. From each selected city, five districts or counties were then randomly chosen. Within each district or county, schools were categorized into rural, township, and urban types, and cluster sampling was conducted accordingly.

From each school, two classes per grade were randomly selected for participation. Inclusion criteria comprised: (1) students enrolled for at least one full semester; (2) students capable of participating normally in physical activities; and (3) students who provided informed consent signed by parents/guardians. Exclusion criteria included: (1) students with physical illnesses or psychological disorders; (2) those whose parents/guardians declined participation; and (3) questionnaires with more than 10% missing data. Ultimately, 49,998 valid questionnaires were obtained. The study strictly adhered to the Declaration of Helsinki, respecting participants' rights and freedoms. Informed consent was obtained both before and after testing. The study protocol received ethical approval from the Academic Committee Ethics Review Board of Chengdu Sports University (Approval No: CDSU2025-139).

### 2.1 Research instruments

#### 2.1.1 Physical activity scale

This study adapted the CDC-YRBS 7-day moderate-to-vigorous physical activity (MVPA) scale (32). The threshold was adjusted to a daily standard of ≥2 h of MVPA in line with China's policy, using a day-by-day recall method for coding. Participants reported whether they had accumulated ≥2 h of MVPA on each of the past 7 days, which was then summarized into the number of weekly days meeting the standard. To align with the policy emphasis on consistent daily implementation in schools, we operationally defined compliance as achieving ≥2 h on ≥4 days per week. Respondents reporting 0–3 days were categorized as non-compliant (coded as 1), while those reporting ≥4 days were categorized as compliant (coded as 0). Considering potential bias from strict daily adherence to the 2-h requirement, corresponding robustness checks were conducted in the statistical analysis. The consistency reliability across the seven daily measurements was 0.78, and the overall convergent validity for key indicators such as frequency of sports participation and adequacy of physical education classes met the standards, with urban–rural differences being significant at *p* < 0.01.

#### 2.1.2 Insufficient sports participation

Drawing on the “frequency of organized or moderate-to-vigorous physical activity” item from GSHS/YRBS (33), participants reporting < 3 sessions of MVPA in the past week were coded as 1; otherwise, they were coded as 0. The number of observed compliance days in the sample was significantly correlated with the number of MVPA compliance days (*r* = 0.41, *p* < 0.001), while the correlation with the proportion of physical education classes was *r* = 0.28. The overall convergent validity was aligned in direction.

#### 2.1.3 Lack of school policy implementation

Referencing the school policy/environment assessment framework from SHPPS/S-PAPA (e.g., designated midday/after-school activity periods, available facilities/equipment) (34), schools were assessed based on actual conditions (Yes = 1, No = 0). The validity of the questionnaire was examined through translation, back-translation, and expert evaluation. A pilot test was conducted with a small sample across three schools. The subscale demonstrated an internal consistency of α = 0.81, and its correlations with variables such as sports equipment availability and class time proportion ranged from 0.35 to 0.49, indicating good convergent validity.

#### 2.1.4 Variable integration

The three items were summed (range 0–3). A primary dichotomous scoring method was used: if any single item was scored as 1, the participant was classified as having “insufficient daily 2-h physical activity,” coded as PAI-2h = 1. If all items were 0, the code remained 0. A split-half reliability test was conducted on the integrated sample, yielding a Spearman-Brown coefficient of 0.72. The measure showed a moderate negative correlation with daily compliance of ≥2 h, which was consistent in direction and statistically significant.

### 2.2 Independent variables (exposure/determinant factors)

#### 2.2.1 Risk factor exposure index (range 0–3)

Defined according to the WHO-GSHS behavioral risk factors module (35). One point was assigned for each of the following: (1) Screen-based sedentary time (non-educational screen time >2 h/day; 2) Sugar-sweetened beverage consumption (≥1 bottle/day; 3) Passive smoking exposure (≥1 day in the past week). Scores were summed (0–3), with higher scores indicating greater exposure. The overall reliability of the scale meets acceptable standards. It shows a positive correlation with the key outcome variable—daily adherence to the ≥2-h threshold—which is both directionally consistent and statistically significant.

#### 2.2.2 Academic stress exposure

Measured using the abbreviated Educational Stress Scale for Adolescents (ESSA) (36) (5 items, Likert 1–5; e.g., burden of learning tasks, time pressure, exam anxiety). The mean score was used, with higher scores indicating greater stress. The scale demonstrates good reliability among Chinese adolescents, with an internal consistency coefficient of α = 0.86. It exhibits a moderate, positive, and statistically significant correlation with the primary outcome variable of daily ≥2-h compliance, supporting its convergent validity.

#### 2.2.3 Lack of facilities/equipment (school environment)

Assessed using items adapted from the S-PAPA/SHPPS school physical activity environment and facility availability scales (3 items: insufficient space, outdated/inadequate equipment, high crowding) (37) (Likert 1–5). Higher mean scores indicated greater resource scarcity. The cultural adaptation and validity of the scale were established through a rigorous process involving translation, back-translation, and expert review. During the pilot phase, the scale demonstrated an internal consistency coefficient (α) of 0.79. It was also found to have a significant positive correlation with factors such as insufficient sports equipment and inadequate physical education class hours, confirming its good convergent validity.

#### 2.2.4 Insufficient physical education (PE) class hours

Based on the SHPPS class hour monitoring framework (38), the ratio of actual weekly PE hours to the national standard was calculated. A ratio < 1 was classified as “insufficient,” and the deficit in hours was recorded. The scale showed significant positive correlations with key indicators including daily compliance with the ≥2-h standard, equipment availability, and policy implementation, demonstrating good convergent validity.

#### 2.2.5 Health cognition/literacy

Measured using the internationally validated Health Literacy for School-Aged Children (HLSAC-10) questionnaire (39) (Yes/No scoring, total score 0–10). Higher scores indicated better health knowledge/literacy. The cultural adaptation of the scale was validated through a process of translation, back-translation, expert review, and pilot testing with a small sample. The scale demonstrated an internal consistency coefficient (α) of 0.83 and exhibited significant correlations with variables such as physical activity level and awareness of the daily 2-h standard, indicating good convergent validity.

#### 2.2.6 Household digital accessibility (optional covariate)

Assessed using items based on the UNICEF-MICS/ICT household digital access module, recording household ownership of internet-capable devices (Yes/No) and overall home internet availability (Yes/No), explaining contexts like “lack of smart devices”. Following back-translation and item review, the scale was found to be significantly correlated with both the lack of smart devices and other key observed variables, confirming its sound convergent validity.

#### 2.2.7 Other covariates

Gender, grade level, geographic region (East/Central/West China), school location (urban/town/rural), school type (public/private), boarding status (boarding/day student), frequency of reunions with parents.

#### 2.2.8 Scale adaptation procedure

The instruments underwent a translation and adaptation process. Initially, native English-speaking scholars performed forward translation into Chinese. Subsequently, Chinese scholars back-translated the texts. The back-translated versions were compared with the original texts to ensure conceptual equivalence, completeness, and linguistic rigor. The questionnaire underwent a comprehensive development and validation process, including translation, back-translation, expert review, pilot testing with a small sample, and semantic adaptation checks. At the predictive stage, all scale items performed well, demonstrating satisfactory internal consistency and overall convergent validity.

### 2.3 Research procedures

Prior to data collection, the research team established a collaborative working group with clearly defined roles, responsibilities, and timelines. During survey administration, trained researchers explained the purpose and procedures, informed participants about the questionnaire items, and monitored participants' status. Questionnaires were anonymous, and participants could withdraw at any time. Data entry was performed independently by two teams using a double-blind procedure to ensure accuracy and standardization. Subsequently, data matching and verification checks were conducted, including tests for consistency (e.g., assessing measurement invariance where applicable), to ensure data integrity.

### 2.4 Statistical analysis

This study adopts a cross-sectional design and focuses on correlation analysis without making causal inferences. All statistical analyses were performed using SPSS 26.0. A two-tailed *p*-value of < 0.05 was set as the threshold for statistical significance, with 95% confidence intervals reported. Multicollinearity was examined using variance inflation factor (VIF) and tolerance diagnostics, with criteria set at VIF < 2 and tolerance >0.5. Analyses included descriptive statistics and regression modeling. The descriptive analysis involved summarizing demographic variables, conducting group comparisons, and employing non-parametric tests (e.g., Mann–Whitney U, Kruskal–Wallis H) where appropriate. For regression analysis, logistic regression was employed, entering control variables and independent variables in blocks. A sensitivity analysis was performed on the operationalized cutoff for daily MVPA ≥2 h, defined as meeting the criterion on ≥4 days per week (coded as compliant) vs. 0–3 days (coded as non-compliant). Repeated analyses were carried out using this dichotomized outcome variable. Finally, heterogeneity tests were performed by constructing separate models for different subgroups (e.g., by school district) to compare Odds Ratio (*OR*) intervals and explore differences between groups.

## 3 Results and analysis

### 3.1 Basic characteristics of the observed demographic variables

As shown in Table 1, a total of 49,998 valid samples were obtained in this study, with geographical distribution as follows: 12,711 samples (25.42%) from South China, 8,366 (16.74%) from Northwest China, 8,257 (16.51%) from Northeast China, 7,667 (15.33%) from Central China, 5,356 (10.72%) from East China, 5,092 (10.18%) from North China, and 2,549 (5.09%) from Southwest China. In terms of school location, the samples were collected from three types of areas: 24,173 students (48.35%) from urban schools, 18,411 (36.82%) from town schools, and 7,414 (14.83%) from rural schools. Regarding school nature, 45,867 students (91.74%) were from public schools, while 4,131 (8.26%) were from private schools. For accommodation type, 34,645 students (69.29%) were day school students, 12,056 (24.11%) were boarders (excluding weekends), and 3,297 (6.59%) were boarders (including weekends). In terms of school stage, the samples were mainly concentrated in the compulsory education stage: 18,514 students (37.03%) in Grades 3–5, 10,230 (20.46%) in Grades 6–7, 10,077 (20.15%) in Grades 1–2, and 8,432 (16.86%) in Grades 8–9. Additionally, there were 2,190 senior high school students (4.38%) and 555 students from higher vocational colleges (1.11%). Regarding the frequency of reunions with parents, 28,231 students (56.46%) reunited with their parents almost every day, 11,473 (22.95%) reunited weekly, 2,693 (5.39%) reunited monthly, 2,518 (5.04%) reunited quarterly, and 5,083 (10.17%) reunited annually.

**Table 1:** Basic information on demographic variables.

**Category**	**Option**	**Frequency**	**Percentage(%)**	**Cumulative percentage(%)**
Administrative region	Northeast China	8,257	16.51	16.51
	North China	5,092	10.18	26.7
	East China	5,356	10.72	37.41
	Central China	7,667	15.33	52.75
	South China	12,711	25.42	78.17
	Southwest China	2,549	5.09	83.27
	Northwest China	8,366	16.74	100
School location School nature	Urban schools	24,173	48.35	48.35
	Town schools	18,411	36.82	85.17
	Rural schools	7,414	14.83	100
	Public schools	45,867	91.74	91.74
	Private schools	4,131	8.26	100
Accommodation type	Boarding (including weekends)	3,297	6.59	6.59
	Boarding (excluding weekends)	12,056	24.11	30.71
	Day school	34,645	69.29	100
School stage	Grades 1–2	10,077	20.15	20.15
	Grades 3–5	18,514	37.03	57.18
	Grades 6–7	10,230	20.46	77.65
	Grades 8–9	8,432	16.86	94.51
	Senior high school	2,190	4.38	98.89
	Higher vocational education	555	1.11	100
Frequency of reunions with parents	Almost every day	28,231	56.46	56.46
	Weekly	11,473	22.95	79.41
	Monthly	2,693	5.39	84.8
	Quarterly	2,518	5.04	89.83
	Annually	5,083	10.17	100
Total		49,998	100	100

### 3.2 Chi-Square test of homogeneity for observed variables

Table 2 indicates that all stratified observed variables were significantly associated with “insufficient daily 2-h physical activity” (e.g., school location, boarding status, school level, frequency of reunions with parents, and school type; all *p* < 0.001). Specifically, the insufficiency rate was highest in rural schools (30.50%) and lowest in urban schools (25.14%). The rate was higher in private schools (29.41%) than in public schools (26.96%). Regarding boarding status, boarding students had the highest rate (30.03%), compared to 27.88% among day students. By school level, junior high school students showed the lowest rate, with a rebound trend observed in higher grades. Furthermore, a clear gradient was evident based on the frequency of reunions with parents: lower reunion frequency was associated with a higher insufficiency rate (starting from 26.46%), indicating a progressively increasing trend.

**Table 2:** Chi-Square test for inadequate daily 2-h physical activity among primary and secondary school students in china.

**Category**		**Physical inactivity rate (%)**		***χ2***	***p***
		**No**	**Yes**		
School location	Urban schools	18,095 (74.86)	6,078 (25.14)	107.402^***^	< 0.001
	Town schools	13,169 (71.53)	5,242 (28.47)		
	Rural schools	5,153 (69.50)	2,261 (30.50)		
School nature	Public schools	33,501 (73.04)	12,366 (26.96)	11.509^**^	0.001
	Private schools	2,916 (70.59)	1,215 (29.41)		
Accommodation type	Boarding (including weekends)	2,307 (69.97)	990 (30.03)	72.29^**^	< 0.001
	Boarding (excluding weekends)	9,125 (75.69)	2,931 (24.31)		
	Day school	24,985 (72.12)	9,660 (27.88)		
School stage	Grades 1–2	7,346 (72.90)	2,731 (27.10)	192.885^***^	< 0.001
	Grades 3–5	12,952 (69.96)	5,562 (30.04)		
	Grades 6–7	7,617 (74.46)	2,613 (25.54)		
	Grades 8–9	6,518 (77.30)	1,914 (22.70)		
	Senior high school	1,545 (70.55)	645 (29.45)		
	Higher vocational education	439 (79.10)	116 (20.90)		
Frequency of reunions with parents	Almost every day	20,760 (73.54)	7,471 (26.46)	131.801^***^	< 0.001
	Weekly	8,585 (74.83)	2,888 (25.17)		
	Monthly	1,899 (70.52)	794 (29.48)		
	Quarterly	1,761 (69.94)	757 (30.06)		
	Annually	3,412 (67.13)	1,671 (32.87)		

^*^P < 0.05.

^**^P < 0.01.

^***^P < 0.001.

### 3.3 Logistic regression analysis of insufficient daily 2-h activity among the overall student population

As presented in Table 3, Model I examined the effects of demographic variables and lack of sports equipment on insufficient 2-h physical activity. Model II assessed the influence of academic stress exposure, while Model III incorporated demographic variables, academic stress exposure, and lack of sports venues to evaluate their combined effects. Overall, as additional variables were sequentially introduced into the models, the goodness-of-fit remained relatively stable (Nagelkerke *R*^2^ = 0.73-0.74), and the direction of the main effects was largely consistent. The three models all passed the Hosmer–Lemeshow test with statistically significant differences, showed acceptable discriminative ability as indicated by the AUC values, had variance inflation factors (VIF) all below 2, and demonstrated good overall model fit.

**Table 3:** Results of the logistic regression analysis for insufficient daily two-hour activity.

**Category**	**Model I**			**Model II**			**Model III**		
	**Wald** χ^2^	***OR***	**95%** ***CI***	**Wald** χ^2^	***OR***	**95%** ***CI***	**Wald** χ^2^	***OR***	**95%** ***CI***
Region	13.062	1.143	1.063–1.229	4.51	1.081	1.006–1.162	9.332	1.121	1.042–1.206
School location	0.002	0.999	0.962–1.037	5.536	1.045	1.007–1.085	0.001	1.000	0.963–1.039
Boarding status	60.230	0.928	0.911–0.946	69.548	0.924	0.907–0.941	63.601	0.926	0.908–0.943
School level	72.113	1.070	1.053–1.087	95.581	1.081	1.063–1.097	58.833	1.064	1.047–1.081
Lack of sports equipment	186.992	2.398	2.116–2.719				116.475	2.040	1.793–2.322
Lack of sports venues	16.317	1.345	1.165–1.553				1.980	1.113	0.959–1.292
Lack of smart devices	1199.245	2.110	2.023–2.201				1014.918	2.007	1.923–2.095
Insufficiency of physical education class hours	171.198	1.443	1.392–1.501				184.367	1.423	1.373–1.679
Exposure to academic stress	18.751	1.093	1.050–1.138				19.253	1.096	1.052–1.141
Lack of cognition regarding PE				9.071	1.274	1.088–1.492	2.740	1.145	0.975–1.345
Low perceived value				520.228	3.724	3.326–4.169	234.242	2.550	2.262–2.875
Lack of awareness of the 2-h standard				445.409	4.408	3.841–5.059	366.106	3.966	3.444–4.567
−2LogLikelihood	2198.914			2651.704			2892.726		
Nagelkerke*R*^2^	0.73.34			0.73.98			0.74.03		

Model I was the regression model incorporating demographic variables and risk exposure factors against insufficient daily two-hour activity.

Model II included demographic variables and health cognition levels.

Model III was the full model, incorporating demographic variables, risk exposure factors, and health cognition levels.

The full model (Model III) identified several key factors that significantly increased the risk of physical activity insufficiency, including: lack of awareness regarding the 2-h activity standard (*OR* = 3.97), low perceived value of physical activity (*OR* = 2.55), insufficient physical education class hours (*OR* = 1.42), lack of sports equipment (*OR* = 2.04), and lack of smart devices (*OR* = 2.01). Academic stress exposure also emerged as a significant risk factor (*OR* = 1.10). In contrast, lack of sports venues was not statistically significant in the full model. Additionally, the risk of insufficient activity showed a slight but consistent increase with higher school levels (*OR* = 1.06–1.08). Model robustness was examined by comparing compliance defined as ≥4 days/week vs. all 7 days/week. Key risk factors maintained consistent direction and significance with minimal effect size changes. Stability was further confirmed through an ordered logistic model using weekly compliant days (0–7) as an ordinal outcome.

### 3.4 Logistic regression analysis of insufficient daily 2-h activity across different school districts

Table 4 indicates acceptable model fit indices across all regional subgroups. Goodness-of-fit and robustness of the regional models were assessed using the Hosmer-Lemeshow test and AUC values, respectively. Both models demonstrated satisfactory fit and discriminative ability, with the primary observed variables showing consistent directionality and statistical significance. In the full model, the direction of the main effects was generally consistent. The following factors significantly increased the risk of physical activity insufficiency: lack of awareness of the 2-h standard (*OR* = 3.88, 95% *CI*: 2.82–5.34), low perceived value of physical activity (*OR* = 2.43, 95% *CI*: 1.85–3.18), lack of sports equipment (*OR* = 2.28, 95% *CI*: 1.71–3.03), and lack of smart devices (*OR* = 1.78, 95% *CI*: 1.60–1.98). Insufficient physical education class hours also significantly increased the risk (*OR* = 1.69, 95% *CI*: 1.29–2.53).

**Table 4:** Results of the logistic regression analysis for insufficient daily 2-h activity by school district.

**Category**	**Model I**			**Model II**			**Model III**			**Urban vs. town vs. rural OR (*F*; *p*)**
	**Wald**χ^2^	***OR***	**95%CI**	**Wald**χ^2^	***OR***	**95%CI**	**Wald**χ^2^	***OR***	**95%CI**	
Region	2.314	1.103	0.972–1.252	5.016	1.144	1.017–1.287	0.238	1.036	0.899–1.194	(−0.418, 0.675)
School location	1.138	1.034	0.972–1.101	1.945	1.044	0.983–1.108	1.305	0.954	0.879–1.035	(−0.202, 0.839)
Boarding status	6.595	0.964	0.937–0.991	45.228	0.901	0.874–0.929	15.918	0.903	0.859–0.949	(3.151, 0.001)
School level	35.833	1.091	1.060–1.122	6.709	1.031	1.008–1.056	8.592	1.054	1.018–1.092	(2.982, 0.002)
Lack of sports equipment	40.782	1.994	1.614–2.465	44.139	1.974	1.615–2.413	31.956	2.277	1.712–3.028	(0.067, 0.946)
Lack of sports venues	4.77	1.298	1.027–1.639	0.426	1.084	0.851–1.380	0.93	0.852	0.615–1.180	(1.051, 0.293)
Lack of smart devices	578.786	2.182	2.048–2.325	321.07	1.88	1.755–2.015	109.179	1.778	1.596–1.981	(3.142, 0.002)
Insufficiency of physical education class hours	89.243	1.389	1.320–2,474	53.073	1.487	1.401–2.591	39.189	1.693	1.293–2.526	(0.592, 0.114)
Exposure to academic stress	29.379	1.182	1.113–1.255	2.339	1.053	0.986–1.125	0.196	1.024	0.923–1.135	(2.499, 0.012)
Lack of cognition regarding PE	6.751	1.39	1.084–1.782	0.281	1.07	0.834–1.372	0.303	0.895	0.604–1.328	(1.458, 0.144)
Low perceived value	112.164	2.706	2.251–3.253	80.259	2.441	2.008–2.968	41.578	2.427	1.854–3.178	(0.743, 0.457)
Lack of awareness of the 2-h standard	129.417	3.657	2.925–4.572	162.859	4.264	3.413–5.328	69.474	3.880	2.821–5.337	(−0.949, 0.342)
−2LogLikelihood	1438.452			1001.008			454.201			
Nagelkerke *R*^**2**^	0.7566			0.7284			0.7127			

Tests for regional heterogeneity showed no significant overall differences among urban, town, and rural areas. However, significant disparities were observed specifically in the lack of smart devices (*t* = 3.142, *p* = 0.002) and academic stress levels (*t* = 2.499, *p* = 0.012). While the strength of association varied, both boarding status and school level consistently demonstrated significant effects on the prevalence of insufficient daily 2-h physical activity.

## 4 Discussion

This study identified significant associations between physical activity (PA) insufficiency and cognitive, supply-side, and exposure-related factors. The most prominent risk factors were a lack of awareness of the 2-h activity standard and a low perceived value of PA, followed by secondary factors such as a lack of sports equipment, insufficient physical education (PE) class hours, and limited access to smart devices. Demographically, higher prevalence rates were correlated with attending rural or private schools, being a boarding student, and having less frequent reunions with parents. Furthermore, the study confirmed that PA insufficiency was linked to adverse outcomes in physical fitness, eyesight, psychological health, and academic performance. These findings provide quantitative evidence for achieving the daily 2-h activity target through coordinated efforts across policy, school, and family levels.

### 4.1 Disparities in insufficient daily 2-h physical activity across different demographic variables

After controlling for supply, cognitive, and exposure variables, the study confirmed that the demographic structure has an independent effect on achieving the standard of the daily 2-h target. Students from rural areas, private schools, and boarding schools, as well as students with less frequent reunions with their parents, had significantly higher risks of insufficiency. It became increasingly more difficult to reach the standard with higher school levels. In subgroup analyses, the effect sizes of the boarding status and school level were different across regions; however, the directional influence was the same. The independent effect of the urban-rural and regional distinctions weakened in the full model.

Multiple studies indicated that children and adolescents' involvement in extracurricular physical activity is related to their environment (40). Children living with family members have the highest probability of physical activity, followed by children boarding in private residences; the lowest levels were found in children in collective dormitories (41). Although the fitness levels and volume of physical activity of urban students are significantly lower than those of their rural counterparts, with relatively large differences in some fitness indices and the volume of moderate-to-vigorous physical activity per week, the perceived availability of physical activity space is positively correlated with both physical fitness and the level of physical activity for all students (42), which is consistent with the findings of this study.

After the inclusion of several control variables, the effects of school level gradient and boarding type on failure to meet the “2-h daily” standard remained significant, while the independent effect of school urban-rural attribute weakened obviously: higher school level students generally had greater difficulty in meeting the standard, boarding students showed significantly higher risk, and urban-rural differences became less robust. The research results are largely consistent with previous international research regarding urban-rural differences, resource availability, time allocation and values. Scholars believe that family and school are two main influences on participation in physical activity, and that support from family resources has a significant effect on the participation of children and adolescents in physical activity and schoolwork (43). Availability of facilities/equipment, curriculum provision, teacher/peer support, and value perception are all positively associated with physical activity and academic achievement (44). Hierarchical regression showed that the effects of low perceived value and lack of awareness of the “2-h” standard were the largest, and that the effects of lack of equipment and insufficient PE hours were stable. In addition, academic pressure as a time constraint also had a significant effect, and was recently discussed in regard to the relationship between academic stress and physical activity and academics (45).

This study is different from previous research in indicator selection and definition of activity context, thereby yielding more practical meanings. Most international literature focuses on “≥1 h daily” or total activity volume, emphasizing the relationship between total activity and health/cognition/academics (46, 47). This study uses the framework of “2 h of comprehensive daily physical activity”, which corresponds to national policy implementation and covers the entire scenario, including PE classes and breaks, after-school services, off-campus activities, thereby finding implementation challenges for the whole process. The results found that guaranteed PE hours and equipment provision were very important, and that cognition value and consistency of participation were key factors. This result is consistent with the school-based intervention study (48), and has a strong correlation with attitudes toward physical activity (49) and participation levels, and is also consistent with the study on teacher supervision and peer support (50).

The results of this study showed that the effect of the independent variable of urban-rural attribute weakened. When we adopt a socio-ecological approach (42), and consider equipment, class hours, health cognition, values, and academic burden simultaneously, the influence factors of different school levels may transfer more to “resources-time-cognition” rather than the geographical location *per se*. Therefore, when policymakers consider policy implementation and regional planning, they should focus more on specific school levels and regional characteristics, and should not be biased about the effectiveness of policies due to regional differences.

### 4.2 Risk exposure factors and insufficient 2 h of daily physical activity

The study found that resource accessibility and time-related exposures are key barriers constraining students' achievement of the daily 2-h physical activity target. Insufficient equipment supply, shortage of PE class hours, lack of smart device access, and academic pressure were all confirmed to significantly increase the risk of not meeting the standard, while lack of sports venues showed no independent effect in the multivariable-adjusted model. Subgroup analyses by region indicated generally consistent directional effects for these factors, but the influence of lack of smart device access and academic pressure exhibited regional heterogeneity. Only insufficient equipment supply demonstrated a robust risk effect across all regions.

Academic pressure encroaches on discretionary time through homework burden and examination schedules, particularly prominent in higher school levels, manifesting as a persistent, albeit modest, but significant risk under the 2-h standard (45). The association of lack of sports facilities weakened after controlling for equipment and class hours, indicating its impact on behavior was more mediated through the chain of resource accessibility and curriculum implementation (49). Lack of smart device access is not equivalent to lack of physical activity, instead, it may impair the process of behavior maintenance, for example, reducing self-monitoring, goal setting and motivation from peers, further leading to shorter duration of activity. Existing reviews and experimental studies showed that goal feedback and social support through smartphone and wearables would improve the physical activity level (51), consistent with our study.

Unlike the existing research, this study further highlighted the core position of class hours, breaks, after-school period and venue elements for sustainable participation, and the squeezing effect of family academic norm and examination rhythm on time structure. The difference was made at a higher threshold: under the 2-h standard, activity in fragments or one-time activities can hardly contribute to reaching the 2-h target, emphasizing the necessity of continuous supply and complete chain of time.

While the variable orientation was finer, distinguishing the positive role of digital tool from simple screen time, we supposed that it could improve adherence through goal management, process feedback and motivation from peers; The explanatory framework was more complete, juxtaposing cognitive constraint and supply in the same model, discovering their effects, and therefore providing the basis for combined strategy, reorganizing the chain of time structure and digital self-monitoring.

### 4.3 Health cognition levels and insufficient daily 2-h physical activity

This study found lack of health cognition and low perceived value of physical activity were the strongest factors affecting achievement of 2-h standard. If students cannot internalize institutional requirement and intrinsic value of 2-h standard as clear behavioral norms and goals, even with available venues and time, forming stable and long duration of participation is still very difficult. If students fully recognize the value of daily 2-h activity, master methods to scientifically allocate 2-h activity, and understand its physical and mental benefits, the possibility of sustainable participation will be significantly improved.

Previous studies have found positive correlations between physical activity/or physical fitness and cognitive outcomes, and between physical activity/or physical fitness and academic outcomes (52). A school-based physical activity program for high school students (*n* = 120) can improve adolescents' health-related fitness (53). Regular physical activity can improve self-perception, emotion, and cognitive function (43). Teacher support, as an important environmental factor, may enhance students' exercise self-efficacy by creating a good class atmosphere and improving peer norms and role models; physical activity is positively correlated with teacher support sources, and hierarchical regression shows that teacher support can predict physical activity levels (50). These conclusions provide strong theoretical basis and support the key findings of this study.

This study, combined with previous studies, clarifies the chain mechanism linking cognition, environment, and self-efficacy, and recognizes the importance of school and peer environment in the internalization of cognition. The difference lies in the more focused conceptual operationalization: This study operationalizes health cognition into the understanding and endorsement of the 2-h policy standard and the value of physical activity, instead of using general indicators of health knowledge; the situational context is more practice-oriented: cognition is studied in the time structure linking PE classes, breaks, and after-school periods, and it is important to recognize that the transformation of cognition into behavior requires structured time and a peer-supported atmosphere. However, as a cross-sectional study, this research does not establish causal relationships. This provides an operable measurement and analysis framework for longitudinal and intervention studies, and provides an empirical basis for the integration of value education and time governance.

### 4.4 Limitations and future workings

Based on the empirical findings, this study provides an evidence-based foundation for promoting the nationwide implementation of the “daily 2-h” physical activity target. However, several limitations should be acknowledged. First, as a cross-sectional study, the present research only identified correlations between variables; it did not examine potential bidirectional causal relationships, particularly between cognitive and exposure-related factors. Second, the use of self-report questionnaires may introduce common method bias—a systematic limitation inherent to such instruments, though not entirely avoidable. Third, although the measurement scales were translated and culturally adapted, the study did not include cross-national comparisons. Potential cultural differences remain unexplored and represent an important direction for future research.

To address these limitations, future studies should adopt longitudinal designs with multiple time points to clarify causal pathways and interactive effects between independent and dependent variables. Moreover, objective physiological and biochemical measurements obtained through modern laboratory equipment are recommended to complement self-reported data and enhance assessment accuracy. Finally, international collaboration with diverse countries and regions would enable cross-cultural comparative studies, helping to control for cultural variations and inform the effective implementation of the daily 2-h activity policy worldwide.

## 5 Conclusion

In summary, based on a national large-sample survey, this study yields the following findings: (1) Demographic variables are primary factors influencing physical activity participation among primary and secondary school students, with higher risks observed among older students, boarders, and those with less parental involvement; (2) Resource accessibility and time constraints are the main barriers to achieving the daily 2-h physical activity target; (3) Health awareness and perceived physical activity engagement are strongly correlated, and internalized cognition and value recognition are highly associated with daily adherence to the 2-h standard; (4) Access to smart devices and academic pressure show significant differences in their impact on urban and rural students, while shortages in sports equipment remain the most prominent contributing factor.

## Data Availability

The raw data supporting the conclusions of this article will be made available by the authors, without undue reservation.
